# Empowerment approach or burden avoidance? How and when leaders’ after-hours electronic communication expectations influence subordinates’ job crafting

**DOI:** 10.3389/fpsyg.2026.1761645

**Published:** 2026-03-12

**Authors:** Xiong Zheng, Lingling Li, Guanfeng Shi

**Affiliations:** School of Economics and Management, Shihezi University, Shihezi, China

**Keywords:** after-hours electronic communication expectations, approach-oriented job crafting, avoidance-oriented job crafting, organization-based self-esteem, regulatory focus, work intensification

## Abstract

In the digital-intelligent era where digital communication management is widely implemented in enterprises, how subordinates appropriately reshape their jobs in response to leaders’ after-hours electronic communication expectations (LAECE) is a critical issue in organizational management. Grounded in cognitive appraisal theory and regulatory focus theory, this study investigates the mechanisms and boundary conditions through which LAECE influences approach-oriented and avoidance-oriented job crafting. Using an experience sampling method, 124 subordinates were tracked over five consecutive days. Data analysis revealed that LAECE positively influences both approach-oriented and avoidance-oriented job crafting. Organization-based self-esteem and work intensification mediate these relationships, respectively. Promotion focus and prevention focus positively moderate the effects of LAECE on organization-based self-esteem and work intensification, respectively, and further strengthen the respective mediating effects. These findings contribute marginally to bridging the theoretical gap in understanding how subordinates cope with LAECE challenges and offer guidance for implementing appropriate job crafting to adapt to digital work contexts.

## 1 Introduction

The proliferation of digital communication technologies has fundamentally reshaped work boundaries (Shi and Zheng, 2021; Zhao and Jiao, 2023), normalizing the expectation for employees to remain connected after hours (Li et al., 2025; Shi et al., 2023). This phenomenon, termed “Leader After-Hours Electronic Communication Expectations” (LAECE), reflects a stable cognitive perception where subordinates feel compelled to respond to work-related communications beyond formal working hours (Cheng et al., 2023; Li et al., 2022; Liu et al., 2025). While survey data indicate that over 80% of employees engage in such after-hours connectivity, often under pressure, current research lacks a comprehensive understanding of how employees adaptively or defensively reconstruct their roles in response to this pervasive expectation (Yang et al., 2022).

Existing literature on after-hours electronic communication (AEC) predominantly adopts a “short-term event-based” perspective, focusing on immediate outcomes like emotional exhaustion or work–family conflict (Yang et al., 2022; He et al., 2023; Dong et al., 2022). This approach overlooks three critical gaps. First, there is a conceptual shift needed from viewing AEC as discrete “events” to understanding LAECE as a stable psychological “expectation.” Unlike isolated incidents, the constant “on-call” state entails enduring implications for role perception and behavioral strategies that remain under-explored (Li et al., 2022; Liu et al., 2025). Second, the cognitive mechanisms driving heterogeneous employee responses remain a “black box.” Employees are not passive recipients; they actively engage in job crafting—proactively altering work roles and boundaries to align interests and abilities with job demands (Wrzesniewski and Dutton, 2001). This encompasses both approach-oriented dimensions (increasing resources) and avoidance-oriented dimensions (reducing hindering demands) (Bruning and Campion, 2018; Chen et al., 2022). While work context and individual traits are known antecedents (Zhang et al., 2012), how individuals cognitively appraise these contextual changes to determine their crafting direction remains a crucial yet under-examined mediating mechanism (Chen et al., 2022). Specifically, facing the digital transformation represented by LAECE (Li et al., 2024; Piszczek, 2017), it is unclear how subordinates form divergent appraisals that trigger either approach or avoidance crafting (Tian et al., 2020). Third, current frameworks lack integration with motivational traits. Without considering stable individual differences, existing models cannot explain why identical expectations are interpreted as opportunities by some and threats by others (Cheng et al., 2023).

To address these gaps, this study integrates Cognitive Appraisal Theory and Regulatory Focus Theory. According to cognitive appraisal theory, individuals evaluate stimuli based on personal significance and select responses congruent with their traits and preferences (Lazarus and Folkman, 1987; Jiang and Wang, 2022). In the LAECE context, this evaluation bifurcates into two pathways: some subordinates may perceive LAECE as recognition of competence and value (Cheng et al., 2023), thereby enhancing their organization-based self-esteem (OBSE) (Li et al., 2022); this positive appraisal prompts approach-oriented crafting, such as increased proactive behaviors (Ren et al., 2023). Conversely, others may perceive LAECE as an additional job demand (Li et al., 2022) that extends work hours (Cheng et al., 2023) and leads to work intensification (Zhao and Jiao, 2023); this negative appraisal induces avoidance-oriented crafting, such as work withdrawal (Chen et al., 2022). Thus, OBSE and work intensification serve as the dual cognitive mechanisms driving divergent crafting strategies.

**Figure 1:**
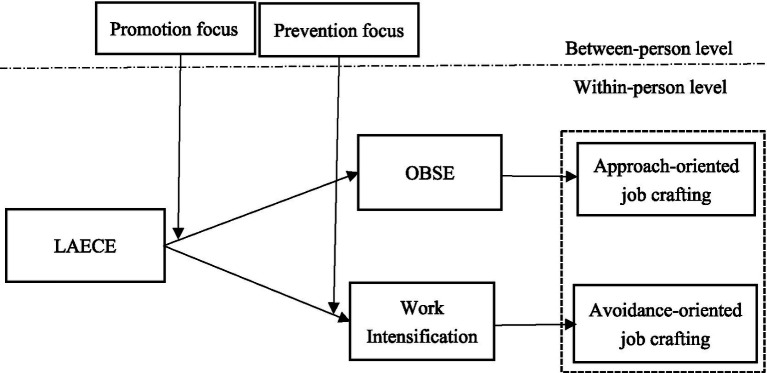
Theoretical model. The model shows LAECE at the within-person level influencing approach-oriented job crafting via organization-based self-esteem, moderated by promotion focus (between-person level), and influencing avoidance-oriented job crafting via work intensification, moderated by prevention focus (between-person level).

By developing a moderated mediation model (Figure 1), this research makes three key theoretical contributions. First, it advances the discourse from “event response” to “expectation management,” extending the theoretical depth of digital work context research by examining the enduring effects of LAECE. Second, it unpacks the “black box” of employee agency by identifying distinct cognitive mediators (OBSE vs. work intensification) that explain the duality of job crafting responses. Third, it delineates precise boundary conditions by integrating regulatory focus, clarifying how motivational traits systematically determine whether LAECE acts as a developmental catalyst or a stressor. Practically, these findings offer nuanced insights for managing the “always-on” culture, suggesting differentiated interventions to guide positive job crafting and foster sustainable career development.

## 2 Theoretical background and hypotheses

### 2.1 LAECE and approach−/avoidance-oriented job crafting

LAECE reflects the extent to which employees perceive their leaders expect them to remain connected for work during non-work hours (Li et al., 2022; Liu et al., 2025), a typical work context change driven by digital technology integration (Shi and Zheng, 2021). Facing this change, employees proactively adjust their boundary relationships with work tasks (Zhao and Jiao, 2023) and adapt through job crafting (Shi et al., 2023). Adaptation strategies vary: some respond proactively (Li et al., 2022), while others react passively or avoidantly (Zhao and Jiao, 2023). This divergence stems from underlying motivational orientations, manifesting as approach-oriented versus avoidance-oriented job crafting (Bruning and Campion, 2018; Chen et al., 2022). The former pursues positive outcomes by expanding job boundaries, enriching social networks, and increasing challenging demands; the latter focuses on preventing losses by simplifying tasks and reducing responsibilities to minimize risks (Wrzesniewski and Dutton, 2001; Zhang and Parker, 2018).

Cognitive appraisal theory provides the core explanatory framework (Lazarus and Folkman, 1987; Jiang and Wang, 2022). If employees appraise LAECE as a challenge or opportunity, they tend towards proactive behaviors; if appraised as a threat, defensive reactions like avoidance ensue (Jiang and Wang, 2022; Zhao et al., 2023). LAECE, being relevant to career development (Li et al., 2022; Liu et al., 2025), triggers evaluations of its value and risk. This heterogeneous cognitive appraisal leads to divergence on the approach-avoidance motivation dimension, resulting in different crafting behaviors (Zhang and Parker, 2018).

From a positive appraisal perspective, employees’ positive appraisal of LAECE motivates them to engage in approach-oriented job crafting. From a resource-gain perspective, promptly responding might be seen as a beneficial investment, expecting reciprocity from the leader (e.g., attention, resources, promotion), fostering high-quality leader-member exchange (Cheng et al., 2023; Ma and Su, 2020). This positive assessment triggers positive emotions (Cheng et al., 2023), promoting proactive behaviors (Ren et al., 2023), like adjusting work-family boundaries for flexibility (Tian et al., 2020) or continuing work after hours (Shi et al., 2023; Chen et al., 2022). From a value-affirmation perspective, even if seen as a challenge, a positive cognitive mechanism can interpret LAECE as leader affirmation of their capability and value (Li et al., 2022; Pierce et al., 1989; Chen et al., 2021), enhancing organization-based self-esteem, competence feelings (Cheng et al., 2023), and potentially satisfying needs for autonomy (Ren et al., 2023), thereby fulfilling basic psychological needs (Shi and Zheng, 2021). This enriched psychological resource pool motivates active adaptation strategies like skill acquisition, feedback-seeking, and process optimization (Shi and Zheng, 2021; Yang et al., 2022; Chen et al., 2022), i.e., approach-oriented job crafting (He et al., 2023).

From a negative cognitive pathway perspective, when employees form negative evaluations of LAECE, it activates their defense mechanisms, leading to avoidance-oriented job crafting. From a threat-conflict perspective, perceiving LAECE as an intrusion into personal life (Shi and Zheng, 2021), foreseeing increased work hours, heightened work–family conflict (Cheng et al., 2023; Li et al., 2022), and impaired family harmony generates strong threat perception, leading directly to avoidance-oriented crafting. From a resource-protection perspective, LAECE objectively increases job demands (Zhao and Jiao, 2023), potentially hindering psychological detachment (Shi and Zheng, 2021) and causing continuous resource depletion (Li et al., 2022). According to conservation of resources theory, perceiving resource loss threat activates protection mechanisms (Shi and Zheng, 2021), prompting withdrawal, avoidance, and refusal behaviors (Zhao and Jiao, 2023; Shi et al., 2023) to prevent further loss. Under this negative appraisal, LAECE is seen as a hindrance stressor (Li et al., 2022), leading employees to reduce resource investment (Cheng et al., 2023) and adopt coping strategies, withdrawal responses, manifesting as avoidance-oriented job crafting characterized by reducing demands (Li et al., 2024; Zhao et al., 2023).

Thus, LAECE may trigger approach or avoidance motivation, leading to corresponding crafting behaviors. We hypothesize:

*H1*: LAECE positively influences subordinates’ approach-oriented job crafting.

*H2*: LAECE positively influences subordinates’ avoidance-oriented job crafting.

### 2.2 The mediating role of organization-based self-esteem

Organization-based self-esteem (OBSE) represents a cognitive appraisal of the extent to which individuals feel their self-worth and significance are validated, grounded in their role experiences within the organizational context (Pierce et al., 1989). This construct emerges from individuals’ specific experiences and role enactments within the organization, manifesting as a positive cognitive appraisal formed by employees following organizational recognition of their personal capabilities (Chen et al., 2021). As a critical psychological resource (Li et al., 2022), organizational self-esteem fosters employees’ willingness to support change and proactive behaviors (Xu and Wang, 2022). Its level is significantly shaped by job characteristics, organizational contexts, and leadership styles (Feng et al., 2024; Chen et al., 2021), such as high-performance work systems, leadership developmental feedback, and differential leadership (Feng et al., 2024; Chen et al., 2021).

Theoretically, LAECE can enhance OBSE through several mechanisms. First, after-hours electronic communication, as a form of high-performance work practice (Cheng et al., 2023; Liu et al., 2025), signals leader trust in the subordinate’s capability (Li et al., 2022). Based on social exchange reciprocity, this trust perception enhances self-worth feelings, raising OBSE (Feng et al., 2024; Zhang et al., 2023). Second, leaders often contact competent, trustworthy subordinates for after-hours liaison regarding critical tasks (Wang and Huang, 2019), creating a differential leadership pattern (Ma and Su, 2020). Subordinates included in the “in-group” through such differential treatment gain higher organizational identification and value affirmation (Chen et al., 2021). Finally, from a basic psychological needs perspective, LAECE offers opportunities to demonstrate autonomy and competence (Shi and Zheng, 2021). Frequent leader-subordinate interaction helps build high-quality leader-member exchange, satisfying needs for autonomy, competence, and relatedness (Cheng et al., 2023), promoting self-actualization, ultimately enhancing OBSE (Chen et al., 2021).

According to cognitive appraisal theory, elevated OBSE promotes approach-oriented job crafting (Lazarus and Folkman, 1987; Jiang and Wang, 2022). High OBSE indicates a positive appraisal of the LAECE situation, triggering approach motivation, prompting crafting responses to leader expectations (Li et al., 2022; Chen et al., 2021). High OBSE serves as an important psychological resource (Li et al., 2022), motivating proactive behavior (Kong et al., 2020), enabling active resource mobilization to meet demands. Furthermore, high OBSE employees identify more with organizational goals, willing to invest more effort (Feng et al., 2024), tending towards role expansion via broadening boundaries and undertaking challenging tasks (Zhang et al., 2023; Chen et al., 2021; Kim and Beehr, 2022), exhibiting typical approach-oriented job crafting.

Thus, We hypothesize:

*H3*: Organization-based self-esteem mediates the relationship between LAECE and approach-oriented job crafting.

### 2.3 The mediating role of work intensification

Work Intensification refers to the employees’ perception of accelerated work pace and extended working hours (Zhao and Jiao, 2023), rather than a resultant state of resource depletion (e.g., fatigue or exhaustion) (Zhao and Wang, 2019). Unlike resource depletion, which denotes an outcome on the “resource side,” work intensification represents an appraisal on the “demand side” (i.e., perceived increased demands) (Li et al., 2022). Therefore, work intensification is a cognitive appraisal, whereas exhaustion is a psychological state outcome. This study focuses on subordinates’ perceptions of LAECE’s potential impact, thus considering work intensification primarily in terms of extended time.

Theoretically, LAECE triggers work intensification via: First, as a special demand diffusing from work to non-work domains (Li et al., 2022), LAECE requires sustained cognitive and time resource investment (Cheng et al., 2023). Frequent switching between work-rest states depletes psychological resources (Li et al., 2022; Liu et al., 2025). Merely monitoring LAECE constitutes an additional job demand (Cheng et al., 2023), prolonging psychological work time (Shi and Zheng, 2021), forming work intensification (Zhao and Jiao, 2023). Second, LAECE demands sustained vigilance for work information (Li et al., 2022). To avoid missing important messages, employees frequently check notifications off-hours, reducing psychological recovery and leisure time (Shi and Zheng, 2021; Liu et al., 2025), causing work time extension (Zhao and Jiao, 2023), strengthening work intensification perceptions.

Per cognitive appraisal theory, work intensification as a negative appraisal triggers avoidance-oriented job crafting (Zhao and Jiao, 2023). When a situation is appraised as threatening, defensive coping strategies are adopted (Lazarus and Folkman, 1987; Jiang and Wang, 2022). Work intensification demands continuous off-hours investment (Shi and Zheng, 2021), leading to excessive resource depletion (Zhao and Jiao, 2023). Specifically, LAECE prolongs psychological work time, causing work intensification (Wrzesniewski and Dutton, 2001), potentially depleting resources and causing work–family conflict (Liu et al., 2025). Appraising work intensification as threatening and feeling resource-deficient generates stress and negative emotions (Lazarus and Folkman, 1987; Jiang and Wang, 2022), leading to withdrawal/avoidance behaviors (Zhao and Jiao, 2023). To conserve finite resources, individuals reduce work investment (Wrzesniewski and Dutton, 2001), responding to demands by avoiding demands/reducing effort (Zhao and Jiao, 2023; Li et al., 2022), exhibiting typical avoidance-oriented job crafting (Chen et al., 2022).

Thus, We hypothesize:

*H4*: Work intensification mediates the relationship between LAECE and avoidance-oriented job crafting.

### 2.4 The moderating role of regulatory focus

Regulatory focus theory distinguishes promotion focus (attaining aspirations/gains) from prevention focus (fulfilling responsibilities/non-losses) (Higgins, 1997; Higgins et al., 2001). This trait influences individuals’ benefit/loss cognitions regarding stimuli (Xu et al., 2022), shapes cognitive appraisal processes in work contexts (Feng et al., 2024), and leads to different behavioral choices (Tian et al., 2020).

Promotion focus strengthens LAECE’s positive effect on OBSE. Promotion-focused individuals are more sensitive to growth-related information (Xu et al., 2022), more likely to interpret LAECE positively (Higgins et al., 2001) as signaling leader trust and value affirmation (Feng et al., 2024), thereby enhancing OBSE (Li et al., 2022). Leaders selecting capable subordinates for contact (Li et al., 2022; Wang and Huang, 2019) enhances self-efficacy more for high-promotion-focused individuals (Cheng et al., 2023), further boosting OBSE (Chen et al., 2021). Moreover, to satisfy value realization needs, high-promotion-focused individuals are more likely to interpret LAECE as competence affirmation (Li et al., 2022), strengthening perceived organizational value (Feng et al., 2024). Therefore, facing identical LAECE, high (vs. low) promotion-focused subordinates perceive higher OBSE.

*H5*: Promotion focus positively moderates the relationship between LAECE and organization-based self-esteem.

Prevention focus the impact of LAECE on job intensification. Individuals with a prevention focus trait tend to concentrate on the depletion of personal resources (Higgins et al., 2001) and are more likely to perceive such expectations as excessive resource consumption (Tian et al., 2020).

Prevention focus strengthens LAECE’s effect on work intensification. Prevention-focused individuals tend to focus on resource depletion (Higgins et al., 2001), and are more likely to perceive this expectation as excessive resource consumption (Tian et al., 2020). Consequently, when confronted with the stimulus of LAECE, they experience a heightened perception of job intensification. Specifically, high-prevention-focused subordinates, focusing on negative aspects, concentrate on potential losses/threats (Feng et al., 2024), e.g., believing responding after hours reduces recovery time (Shi and Zheng, 2021), decreases family domain resources (Liu et al., 2025), further depletes personal resources (Li et al., 2022), leading to perceptions of longer hours/higher intensity (Zhao and Wang, 2019). Therefore, when facing the same LAECE, subordinates with high prevention focus traits are more likely than those with low prevention focus traits to perceive such expectations as an increase in job demands and an extension of working hours. This perception leads to a greater potential threat of accelerated resource drain for these subordinates (Li et al., 2022), resulting in a higher perceived level of work intensification.

*H6*: Prevention focus positively moderates the relationship between LAECE and work intensification.

Furthermore, promotion focus moderates OBSE’s mediating role. High-promotion-focused individuals focus more on resource acquisition (Higgins et al., 2001), more likely viewing LAECE as a pathway to gain trust and achieve work autonomy (Cheng et al., 2023; Li et al., 2022), strengthening the motivation for self-value realization via OBSE (Chen et al., 2021). Per cognitive appraisal theory (Lazarus and Folkman, 1987), this positive expectation prompts active responses to leader expectations (Li et al., 2022; Chen et al., 2021), exhibiting more approach-oriented crafting. Research shows employees perceiving trust and high OBSE reciprocate via role expansion (Zhang et al., 2023) and other approach-oriented crafting (Chen et al., 2021; Kim and Beehr, 2022).

*H7*: Promotion focus positively moderates the indirect effect of LAECE on approach-oriented job crafting via organization-based self-esteem.

Simultaneously, prevention focus moderates work intensification’s mediating role. Prevention-focused individuals highly prioritize resource protection (Higgins et al., 2001), more easily perceiving LAECE as a resource depletion threat (Li et al., 2022; Tian et al., 2020), generating stronger work intensification perception. Even when responding necessarily, high-prevention-focused individuals still experience strong work intensification; accelerated resource consumption prompts protective strategies (Li et al., 2022), reducing proactive behaviors (Chen et al., 2022), decreasing work investment (Wrzesniewski and Dutton, 2001), ultimately exhibiting work withdrawal and other avoidance-oriented crafting (Tian et al., 2020; Zhao et al., 2023).

*H8*: Prevention focus positively moderates the indirect effect of LAECE on avoidance-oriented job crafting via work intensification.

## 3 Research design

### 3.1 Method and sample

A daily diary survey was conducted over five consecutive workdays using experience sampling methodology. Participants were recruited via the research team’s social networks. For geographical dispersion, temporary anonymous We-Chat APP survey groups were created. Participants provided demographic information and regulatory focus measures initially. Pre-designed survey links were distributed at different times daily: the morning survey (LAECE, OBSE, Work Intensification) was sent at 7:00 a.m., requiring completion by 9:00 a.m. regarding the previous day; the evening survey (Approach/Avoidance Job Crafting) was sent at 7:00 p.m., requiring completion by 9:00 p.m. All coded questionnaires were matched at the individual level.

Initially, 150 employees from 8 finance and IT service firms in Guangzhou, Dongguan, Nanchang, and Guiyang participated. After excluding participants with fewer than three days of valid responses or unmatched surveys, the final sample comprised 124 participants yielding 612 daily observations (81.6% valid response rate). Among the 124 participants, 74 were female, age ranged from 21 to 56 (M = 29.77, SD = 6.73), tenure with current leader ranged from 0.25 to 10 years (M = 2.72, SD = 4.17), weekly work hours ranged from 40 to 60 (M = 46.88, SD = 7.31), and 79.0% were non-supervisory staff.

### 3.2 Measures

Established scales were adapted contextually (e.g., adding “in the past day”) for daily assessment (Shi et al., 2023; Li et al., 2022; Ren et al., 2023). Non-English scales underwent translation-back-translation. All items used 5-point Likert scales (1 = strongly disagree, 5 = strongly agree).

LAECE: Adapted from Li et al. (2022), 8 items (e.g., “In the past day, my leader expected me to respond promptly to messages during non-work hours”) (*α* = 0.93).

OBSE: Adapted from Pierce et al. (1989) via Li et al. (2022), 10 items (e.g., “My leader’s after-hours communication made me feel I am valuable in the organization”) (α = 0.91).

Work Intensification: Used the extended working time dimension from Zhao and Wang’s scale (Zhao and Wang, 2019), 3 items (e.g., “My leader’s after-hours communication made me feel that handling work affairs at home is increasingly common”) (α = 0.83).

Job Crafting: Adapted from Bruning and Campion (2018). Approach-oriented (5 items, e.g., “I expanded my work content to obtain resources facilitating task completion”, α = 0.85). Avoidance-oriented (7 items, e.g., “I found ways to avoid time-consuming work”, α = 0.87).

Regulatory Focus: Used Zhou et al.’s scale (Zhou et al., 2012). Promotion focus (4 items, e.g., “I frequently think about how to achieve my work goals”, α = 0.84). Prevention focus (3 items, e.g., “I am more inclined to avoid losses than achieve gains”, α = 0.82).

Control Variables: Following Li et al. (2022), controlled for gender, age, education, marital/parental status, and leader tenure.

## 4 Data analysis and results

Analyses used Mplus 7.4 for multilevel modeling. Except for controls and regulatory focus (between-person), all focal variables were within-person.

### 4.1 Descriptive statistics and correlations

Table 1 shows means, standard deviations, and correlations. LAECE correlated positively with OBSE (r = 0.237, *p* < 0.01), work intensification (r = 0.312, *p* < 0.01), approach-oriented crafting (r = 0.214, *p* < 0.01), and avoidance-oriented crafting (r = 0.389, *p* < 0.01). OBSE correlated positively with approach-oriented crafting (r = 0.451, *p* < 0.01). Work intensification correlated positively with avoidance-oriented crafting (r = 0.517, *p* < 0.01). These preliminary results support hypotheses.

**Table 1:** Means, standard deviations, and correlations.

Variables	M	SD	1	2	3	4	5	6	Within-person variance component
Within-person									
1. LAECE	3.81	0.70							62.81%
2. OR	3.27	0.56	0.237**						31.67%
3. WO	3.39	0.61	0.312**	0.167**					48.06%
4. AP	3.17	0.82	0.214**	0.451**	−0.102*				42.31%
5. AV	3.34	0.86	0.389**	−0.136*	0.517**	−0.491**			39.84%
Between-person									
1. GE	1.40	0.49							
2. AG	29.77	6.73	−0.087						
3. ED	2.46	0.85	−0.147	−0.118*					
4. MA	1.51	0.51	0.134	0.078	0.051				
5. WT	2.72	4.17	−0.021	0.286**	0.048	−0.035			
6. PRO	3.87	0.78	0.317**	−0.120*	0.141*	−0.034	0.061		
7. PRE	2.83	0.64	0.047	0.118*	−0.013	0.061	−0.046	−0.091	

OR, WO, AP, AV, GE, AG, ED, MA, WT, PRO, and PRE denote OBSE, work intensification, approach-oriented job crafting, avoidance-oriented job crafting, gender, age, education level, marital and parental status, working tenure, promotion focus, and prevention focus, respectively; *p < 0.05, **p < 0.01.

Variance decomposition showed within-person variance percentages ranged from 31.67 to 62.81%, all >10%, justifying multilevel analysis.

Multilevel confirmatory factor analysis indicated the hypothesized 7-factor model fit best (χ^2^/df = 2.143, CFI = 0.913, TLI = 0.906, RMSEA = 0.056), supporting discriminant validity.

### 4.2 Hypothesis testing

Within-person predictors were group-mean centered; regulatory focus was grand-mean centered. Monte Carlo simulation (5,000 reps) estimated 95% CIs.

Main effects and mediation: Table 2 shows LAECE positively predicted approach-oriented (*β* = 0.156, *p* < 0.001) and avoidance-oriented (β = 0.312, *p* < 0.001) job crafting, supporting H1 and H2. When LAECE and OBSE (or work intensification) were included together, both coefficients remained significant for their respective outcomes, and LAECE positively predicted OBSE and work intensification (Table 3, β = 0.198, *p* < 0.001; β = 0.127, *p* < 0.001), suggesting mediation. Monte Carlo results (Table 4) confirmed significant indirect effects via OBSE (Estimate = 0.049, 95%CI [0.021, 0.109]) and work intensification (Estimate = 0.097, 95%CI [0.039, 0.198]), supporting H3 and H4.

**Table 2:** Multilevel regression results for main and mediation effects.

Variables	AP				AV			
	β	SE	β	SE	β	SE	β	SE
Intercept	3.714***	0.528	2.152***	0.616	3.217***	0.517	2.279***	0.542
GE	0.210	0.152	−0.104	0.176	−0.037	0.163	0.117	0.211
AG	0.216**	0.081	0.154	0.112	0.146*	0.131	0.155	0.108
ED	0.110	0.148	0.112	0.143	0.059	0.167	0.168	0.234
MA	0.221**	0.064	0.164	0.986	−0.154	0.084	0.142	0.176
WT	0.189*	0.074	0.128	0.091	0.152	0.096	0.147	0.094
LP	0.156***	0.037	0.107**	0.034	0.312***	0.075	0.215***	0.056
OR			0.387***	0.086				
WO							0.489***	0.093
R^2^_within_	0.316	0.041	0.368	0.046	0.327	0.052	0.334	0.054
R^2^_between_	0.341	0.044	0.412	0.051	0.361	0.073	0.426	0.093

OR, WO, AP, AV, GE, AG, ED, MA, WT, PRO, and PRE denote OBSE, work intensification, approach-oriented job crafting, avoidance-oriented job crafting, gender, age, education level, marital and parental status, working tenure, promotion focus, and prevention focus, respectively; *p <0.05, **p < 0.01, ***p < 0.001.

**Table 3:** Multilevel regression results for moderation effects.

Variables	OR				WO			
	β	SE	β	SE	β	SE	β	SE
Intercept	1.775***	0.524	1.632***	0.513	1.881***	0.517	1.872***	0.512
GE	0.118	0.163	−0.152	0.157	0.046	0.105	0.106	0.314
AG	0.186*	0.097	0.154*	0.110	0.121	0.094	0.152	0.101
ED	0.104	0.127	0.101	0.131	0.088	0.217	0.183	0.223
MA	0.103	0.088	0.114	0.086	0.221**	0.147	0.167*	0.154
WT	−0.168*	0.093	0.102	0.089	0.146	0.093	0.122	0.094
LP	0.127***	0.034	0.113**	0.031	0.198***	0.061	0.135***	0.058
PRO			0.317***	0.054				
PRE							0.329***	0.092
LP* PRO			0.214***					
LP* PRE							0.237***	

OR, WO, AP, AV, GE, AG, ED, MA, WT, PRO, and PRE denote OBSE, work intensification, approach-oriented job crafting, avoidance-oriented job crafting, gender, age, education level, marital and parental status, working tenure, promotion focus, and prevention focus, respectively; *p <0.05, **p < 0.01, ***p < 0.001.

**Table 4:** Results for indirect, moderation, and moderated mediation effects.

Effect type	Path/condition	Estimate	SE	95%CI low	95%CI high
Indirect effect	LP → OR→AP	0.049	0.021	0.021	0.109
	LP → WO → AV	0.097	0.042	0.039	0.198
Moderation effect	LP → OR (+1SD)	0.218	0.091	0.043	0.176
	LP → OR (-1SD)	0.035	0.016	−0.054	0.114
	LP → OR (Difference)	0.183	0.083	0.007	0.189
	LP → WO (+1SD)	0.371	0.053	0.061	0.197
	LP → WO (-1SD)	0.026	0.067	−0.06	0.102
	LP → WO (Difference)	0.345	0.058	0.011	0.202
Moderated mediation	LP → OR→AP (+1SD)	0.083	0.039	0.004	0.153
	LP → OR→AP (-1SD)	0.005	0.010	−0.036	0.141
	LP → OR→AP (Difference)	0.078	0.033	0.005	0.189
	LP → WO → AV (+1SD)	0.172	0.043	0.052	0.174
	LP → WO → AV (-1SD)	0.036	0.034	−0.008	0.092
	LP → WO → AV (Difference)	0.136	0.038	0.011	0.145

**Figure 2:**
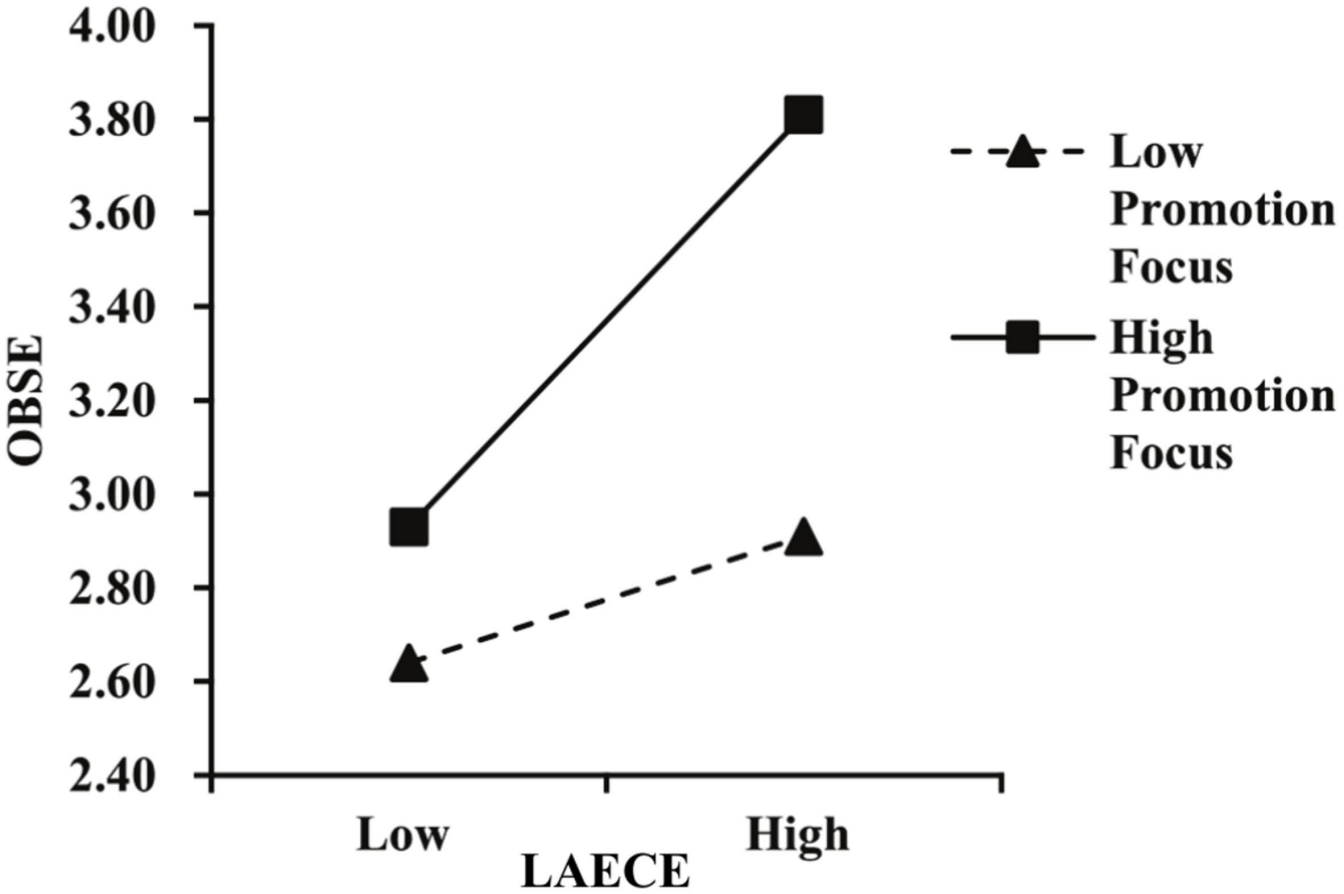
The moderating effects of promotion focus on LAECE and OBSE.

**Figure 3:**
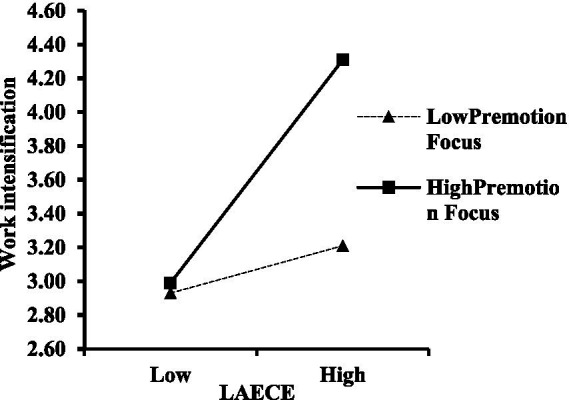
The moderating effects of premotion focus on LAECE and work intensification.

Moderation: Table 3 shows LAECE × Promotion Focus interaction significantly predicted OBSE (β = 0.214, *p* < 0.001), and LAECE × Prevention Focus interaction significantly predicted work intensification (β = 0.237, *p* < 0.001). Simple slope plots (Figures 2, 3) confirmed the interactions. Monte Carlo (Table 4) showed significant conditional effects at high (+1 SD) but not low (−1 SD) levels of the moderators, and significant differences between high and low levels, supporting H5 and H6.

Moderated mediation: Monte Carlo results (Table 4) showed the indirect effect of LAECE on approach-oriented crafting via OBSE was significant at high (+1 SD) promotion focus (Estimate = 0.083, 95%CI [0.004, 0.153]) but not at low (−1 SD) (Estimate = 0.005, 95%CI [−0.036, 0.141]), with a significant difference (Estimate = 0.078, 95%CI [0.005, 0.189]), supporting H7. Similarly, the indirect effect on avoidance-oriented crafting via work intensification was significant at high (+1 SD) prevention focus (Estimate = 0.172, 95%CI [0.052, 0.174]) but not at low (−1 SD) (Estimate = 0.036, 95%CI [−0.008, 0.092]), with a significant difference (Estimate = 0.136, 95%CI [0.011, 0.145]), supporting H8.

## 5 Discussion

### 5.1 Theoretical contributions

First, this study shifts the theoretical lens from discrete “communication events” to stable “connectivity expectations.” Prior research has largely treated after-hours communication as isolated stressors triggering immediate resource depletion (Shi and Zheng, 2021; Zhao and Jiao, 2023). By conceptualizing LAECE as a persistent psychological expectation (Li et al., 2022), we extend the temporal scope of digital work research. This perspective captures the chronic nature of the “always-on” culture, offering a more robust explanation for how sustained leader expectations reshape employees’ long-term role definitions and behavioral strategies, rather than merely inducing transient fatigue.

Second, we elucidate the dual cognitive mechanisms underlying heterogeneous job crafting responses (Zhao and Jiao, 2023). Moving beyond unidimensional outcome models, our findings reveal that LAECE simultaneously activates opposing cognitive appraisals: an empowering pathway through enhanced OBSE and a burdening pathway through perceived work intensification. This duality resolves the theoretical puzzle of why the same contextual factor can lead to both proactive role expansion and defensive withdrawal. By empirically validating these parallel mediators, we enrich Cognitive Appraisal Theory within the digital transformation context, demonstrating that employee agency is driven by the interplay of self-evaluative and demand-based assessments.

Third, we achieve a theoretical integration of contextual demands and individual motivational traits. Previous studies often examined contextual antecedents or individual traits in isolation (Wrzesniewski and Dutton, 2001; Chen et al., 2022; Li et al., 2024). By introducing Regulatory Focus Theory as a boundary condition (Piszczek, 2017), we clarify when and for whom LAECE functions as a challenge versus a threat. Our results demonstrate that promotion and prevention focuses act as perceptual filters, systematically directing employees toward distinct cognitive pathways and subsequent crafting behaviors. This integration not only expands the applicability of Regulatory Focus Theory to digital work settings but also provides a nuanced framework for predicting individual differences in adapting to technology-induced work demands.

### 5.2 Practical implications

Our findings offer several nuanced insights for organizations and leaders navigating the “always-on” digital work context. Given the observational nature of our diary study, the following suggestions should be viewed as potential strategies derived from observed associations rather than definitive causal prescriptions for management interventions.

First, leaders need to recognize the dual-edged sword of Leader After-Hours Electronic Communication Expectations (LAECE). Our results indicate that LAECE can simultaneously trigger both approach crafting (via enhanced OBSE) and avoidance crafting (via perceived work intensification). Rather than simply abolishing after-hours communication, leaders are advised to cultivate awareness of how their expectations are cognitively appraised by subordinates. Excessive or ambiguous expectations may inadvertently signal resource depletion, prompting employees to withdraw or resist change. Therefore, leaders should strive for clarity and moderation in digital communication, aiming to frame after-hours connectivity as an opportunity for role expansion rather than an uncontrollable burden.

Second, management strategies should be tailored to employees’ regulatory focus. The data suggests that the impact of LAECE is not uniform but depends on individual traits. For employees with a high promotion focus: LAECE appears to align with their needs for growth and achievement, potentially enhancing their OBSE. Leaders might consider offering these individuals greater autonomy and challenging tasks through digital channels, leveraging their tendency to view such expectations as opportunities for career development.

For employees with a high prevention focus: these individuals are more likely to perceive LAECE as a threat leading to work intensification and subsequent avoidance behaviors. For this group, leaders are advised to exercise caution. Practical steps may include providing additional resource support, adopting a more supportive emotional tone in messages, and allowing flexible response windows to facilitate recovery.

This differentiation highlights that a “one-size-fits-all” approach to digital leadership is likely ineffective; instead, leaders should adapt their communication styles to mitigate potential negative appraisals among prevention-focused staff.

Finally, employees can play an active role in managing their responses to after-hours demands. While completely disconnecting may not always be feasible, our findings suggest that employees can engage in proactive job crafting to navigate these demands. Rather than passively accepting all requests or rigidly avoiding them, employees are encouraged to assess their current resource levels. When resources are sufficient, moderately engaging in after-hours tasks can be framed as a challenge to build skills and visibility. Conversely, when facing resource scarcity, employees should feel empowered to communicate their boundaries and seek support, transforming potential stressors into manageable challenges. This balanced approach allows employees to maintain career momentum while protecting their well-being from the detrimental effects of constant connectivity.

### 5.3 Limitations and future research

First, although the diary method reduces recall/social desirability bias versus static surveys, common method bias might exist due to single-source self-reports. Future research could use multi-source data (e.g., leader-subordinate dyads). Second, while revealing OBSE and work intensification as mediators, other pathways [e.g., leader trust (Li et al., 2022), personal achievement perception (Gong et al., 2024)] warrant exploration. Finally, this study only examined individual-trait boundary conditions for the mediation paths; future research could explore organizational/family support or other beyond-individual factors moderating LAECE’s impact on different crafting directions and paths.

## 6 Conclusion

Constructing an integrative framework based on cognitive appraisal and regulatory focus theories, this study systematically examined the mechanisms and boundary conditions of LAECE’s impact on subordinates’ job crafting via a dual-path model distinguishing approach and avoidance crafting. Using a daily diary design, the main conclusions are:

First, LAECE exerts a dual influence on subordinates’ job crafting behaviors. Previous-day LAECE levels significantly predict next-day approach-oriented and avoidance-oriented job crafting, confirming the complexity of employee responses in digital work contexts, moving beyond unidimensional views of electronic communication impacts.

Second, OBSE and work intensification play parallel mediating roles. Previous-day LAECE indirectly promotes next-day approach-oriented crafting by enhancing OBSE (positive appraisal path), while indirectly triggering next-day avoidance-oriented crafting by exacerbating work intensification (negative appraisal path). This dual-path mechanism verification comprehensively reveals the psychological process of coping with digital work demands from a cognitive appraisal perspective.

Third, regulatory focus traits play crucial moderating roles. Promotion focus strengthens both LAECE’s positive effect on OBSE and the mediating effect of OBSE between LAECE and approach crafting. Prevention focus amplifies both LAECE’s effect on work intensification and the mediating effect of work intensification between LAECE and avoidance crafting. This finding clarifies the source of individual differences in responding to LAECE from a motivational orientation perspective, refining the “situation-cognition-behavior” transmission mechanism framework.

By constructing and validating a moderated dual-mediation model, this study reveals the internal mechanisms of employee responses to leader connectivity expectations in digital work contexts, advancing research theoretically (integrating cognitive appraisal and regulatory focus theories), methodologically (using diary studies for dynamic capture), and practically (offering targeted implications for managing after-hours electronic communication and guiding positive adaptation).

## Data Availability

The raw data supporting the conclusions of this article will be made available by the authors, without undue reservation.
